# Artificial lamina after laminectomy: Progress, applications, and future perspectives

**DOI:** 10.3389/fsurg.2023.1019410

**Published:** 2023-02-02

**Authors:** Jing Yue, Qing Han, Hao Chen, Aobo Zhang, Yang Liu, Xuqiang Gong, Yang Wang, Jincheng Wang, Minfei Wu

**Affiliations:** ^1^ Department of Anesthesiology, The Second Hospital of Jilin University, Changchun, China; ^2^ Department of Orthopedics, The Second Hospital of Jilin University, Changchun, China

**Keywords:** laminectomy, artificial lamina, epidural fibrosis (EF), finite element analysis, additive manufacturing (3D printing)

## Abstract

In clinical practice, laminectomy is a commonly used procedure for spinal decompression in patients suffering from spinal disorders such as ossification of ligamentum flavum, lumbar stenosis, severe spinal fracture, and intraspinal tumors. However, the loss of posterior column bony support, the extensive proliferation of fibroblasts and scar formation after laminectomy, and other complications (such as postoperative epidural fibrosis and iatrogenic instability) may cause new symptoms requiring revision surgery. Implantation of an artificial lamina prosthesis is one of the most important methods to avoid post-laminectomy complications. Artificial lamina is a type of synthetic lamina tissue made of various materials and shapes designed to replace the resected autologous lamina. Artificial laminae can provide a barrier between the dural sac and posterior soft tissues to prevent postoperative epidural fibrosis and paravertebral muscle compression and provide mechanical support to maintain spinal alignment. In this paper, we briefly review the complications of laminectomy and the necessity of artificial lamina, then we review various artificial laminae from clinical practice and laboratory research perspectives. Based on a combination of additive manufacturing technology and finite element analysis for spine surgery, we propose a new designing perspective of artificial lamina for potential use in clinical practice.

## Introduction

Laminectomy is a widely-applied technique in clinical practice. Lumbar disorders, such as ossification of ligamentum flavum, lumbar stenosis, severe burst fracture, and intraspinal tumor, can cause various spinal compressive symptoms, often requiring spinal canal decompression (1–8). Despite the utility of minimal invasive spinal surgeries, which cause less damage to the posterior part of the spinal column, studies have shown open-door decompressive laminectomy is still an effective method to treat lumbar spinal stenosis (4, 9). Laminectomy can offer adequate space for the spinal cord and provide instant decompression. However, postoperative complications related to laminectomy may cause recurrent symptoms and necessitate secondary surgery. Such complications include iatrogenic spinal instability, epidural fibrosis, and back muscle injury, all of which may cause fail back surgery syndrome (FBSS) (10). The inevitable complications result from lamina resection. Removal of the lamina may lead to lordosis or spondylolisthesis because of biomechanical instability of the spine. In the traditional posterior lumbar approach, paravertebral soft tissues are sutured directly above the dura mater, lack of mechanical barrier between the dura mater and posterior structure can cause proliferation of fibroblast into the spinal cord and result in epidural adhesion/fibrosis. Losing attachment sites for paravertebral muscles/ligaments leads to muscle weakness. Artificial lamina, a synthetic lamina tissue made of various materials and shapes, is intended to replace the resected autologous lamina. Artificial lamina implantation is a straightforward solution to prevent laminectomy-related complications for vertebral reestablishment. Firstly, an artificial lamina is instant structural support to the spine and is an osteogenesis “bridge” for a bony reunion, which is the ultimate purpose of an implant prosthesis (11, 12). Secondly, artificial lamina can be a mechanical barrier between the dura sac and posterior structures, especially those hyperplasia fibroblasts, which can prevent epidural adhesion/fibrosis (13, 14). Finally, artificial lamina can provide attachment sites for paravertebral muscles/ligaments to enhance muscle strength (15).

Clinical practitioners have used various titanium-made prostheses as artificial lamina to reconstruct the posterior column, which provided an instant lamina structure for the stability of the spinal column and protection of the neural elements (16, 17). At the same time, the orthopedic doctors sought to use polymer material to form artificial lamina to prevent epidural fibrosis and found well results. The artificial lamina positively affects epidural fibrosis, but the mechanism of preventing dural adhesion is unclear. Meanwhile, the artificial lamina not only serves as a barrier between the dura and the posterior tissue but also needs to provide mechanical support to the spinal column (18). However, the stress distribution of these artificial laminae remains unclear, considering various materials present different mechanical properties.

With the rapid development of material science and tissue engineering, many researchers have focused on designing materials to prevent epidural fibrosis and induce osteogenesis. Inorganic materials such as alpha-tricalcium phosphates (α-TCP), β-tricalcium phosphate (β-TCP), hydroxyapatite (HA), and mesoporous bioactive glass (MBG) have been incorporated with organic materials to develop inorganic or organic composites. On the one hand, polymeric materials can be used as the physical barrier; on the other hand, polymeric materials can be used as the carrier in the controlled release of chemical drugs (19). Stem cells are seeded on scaffolds as a “bridge” for osteogenesis induction and bony reconstruction of the posterior column (20). To explore the biomechanical effect of the artificial lamina, interdisciplinary medical engineering, such as finite element analysis (FEA) and additive manufacturing (AM), have been introduced. FEA can simulate the real stress situation and predict the biomechanics of implant and bone tissue. Based on FEA, a basic structure composed of finite elements is established in the design space, and then the elements in the designed space are determined according to the algorithm (21–24). The introduction of AM can facilitate customization and better bony reconstruction (25).

This paper briefly introduces the complications of open laminectomy and the necessity of artificial lamina in clinical practice. Then we review the different types of artificial laminae which have already been applied clinically and those still under research. Finally, the advances in the field of additive manufacturing technology and finite element analysis in spine surgery are reviewed, and the future perspective of 3D printing artificial lamina is proposed. This review aims to provide an elementary basis for future artificial lamina investigation and design.

## Complications of laminectomy

Facet-preserving laminectomy is the current gold standard surgical technique for posterior laminectomy. This technique involves a midline lumbar incision and extensive resection of the posterior bone, posterior ligaments, and local muscular structures (26). Despite the prevalence of laminectomy, it may cause several postoperative complications. These complications include iatrogenic instability, epidural fibrosis, and back muscle injury resulting from missing autologous lamina. If treated improperly, some complications may lead to FBSS, persistent radicular and/or lumbar pain and lower extremity pain after spine surgery, which is used to describe a large and diverse group of patients who have undergone a variety of lumbar surgeries with unsatisfactory outcomes (27).

**Iatrogenic spinal instability** is an instability secondary to direct surgical and/or medical intervention. It is radiographically defined as the translational motion of a spinal segment over an adjacent segment at the level of a previous decompression (28). Postoperative iatrogenic instability has been reported at different spine levels, from cervical laminectomy to lumbar spine fusion (29–32). Furthermore, laminectomy for lumbar stenosis can disrupt spinal stability and result in iatrogenic spondylolisthesis in patients with no overt pre-existing instability. Therefore, a substitute for the decompressed bony support can be implanted to preserve spinal integrity and maintain spinal stability. Instead of the rigid lumbar spine after pedicle screw instrumentation, the artificial lamina can provide instant support by sharing the load to help stabilize the spine column.

**Epidural fibrosis** is a common cause of pain after lumbar spine surgery, such as laminectomy, and has been implicated in 8% to over 60% of cases of FBSS (33–36). The exact mechanism of epidural fibrosis is complex and remains unclear. After many years of research, the ventral originated (37)/dorsal originated (38) theory was published. However, the current consensus is the three-dimensional fibrosis formation theory (39). The pathophysiological mechanisms of epidural fibrosis include excessive deposition of extracellular matrix, which includes collagen, fibronectin, and dermatan sulfate, and a decrease in tissue cellularity (40–42). While extensive research on FBSS and epidural fibrosis has been performed in recent years, the best strategy to reduce its incidence and the associated morbidity is to focus on prevention (43). A mechanical barrier between the dural sac and the posterior tissue is an effective method. Artificial lamina made of a specific material can prevent the extensive proliferation of fibroblasts and scar formation.

**Back muscle injury** occurs in all patients who undergo laminectomy because of the incision of the posterior ligaments and muscular structures in the area. The sequelae of posterior surgery include atrophy, loss of cross-sectional area (CSA), fat infiltration, and reduced strength of back muscles (44). Progression of the impairment and disability can lead to severe low back pain, which is an essential cause of FBSS (10, 45). Furthermore, the paraspinal muscles of the lumbar spine may play an important role in the etiology of adjacent segment changes besides a spinal fusion, and this effect is independent of spinal instrumentation (23). Therefore, paraspinal muscle injury may lead to adjacent segment disorder (ASD). Reconstruction of the paravertebral muscles requires a structure for posterior muscle attachment, along with the posterior ramus of the spinal nerve (46). Artificial lamina can provide muscle/ligament attachment sites for paravertebral muscle reconstruction.

Complications of laminectomy have greatly restrained its application in patients requiring posterior decompression. The invention of the artificial lamina is an applicable method. Some artificial laminae have been used in clinical practice, while many are still in the phase of experimental research.

## Artificial lamina applied in clinical practice

Clinical practitioners focused on instant stabilization of the posterior spinal column by different novel types of artificial laminae. Most artificial laminae were applied to associate with posterior lumbar instrumentation fusion (PLIF), while the others were designed to prevent epidural fibrosis simultaneously. Therefore, biomaterial and non-biomaterial have been used to fabricate artificial laminae.

**Figure 1:**
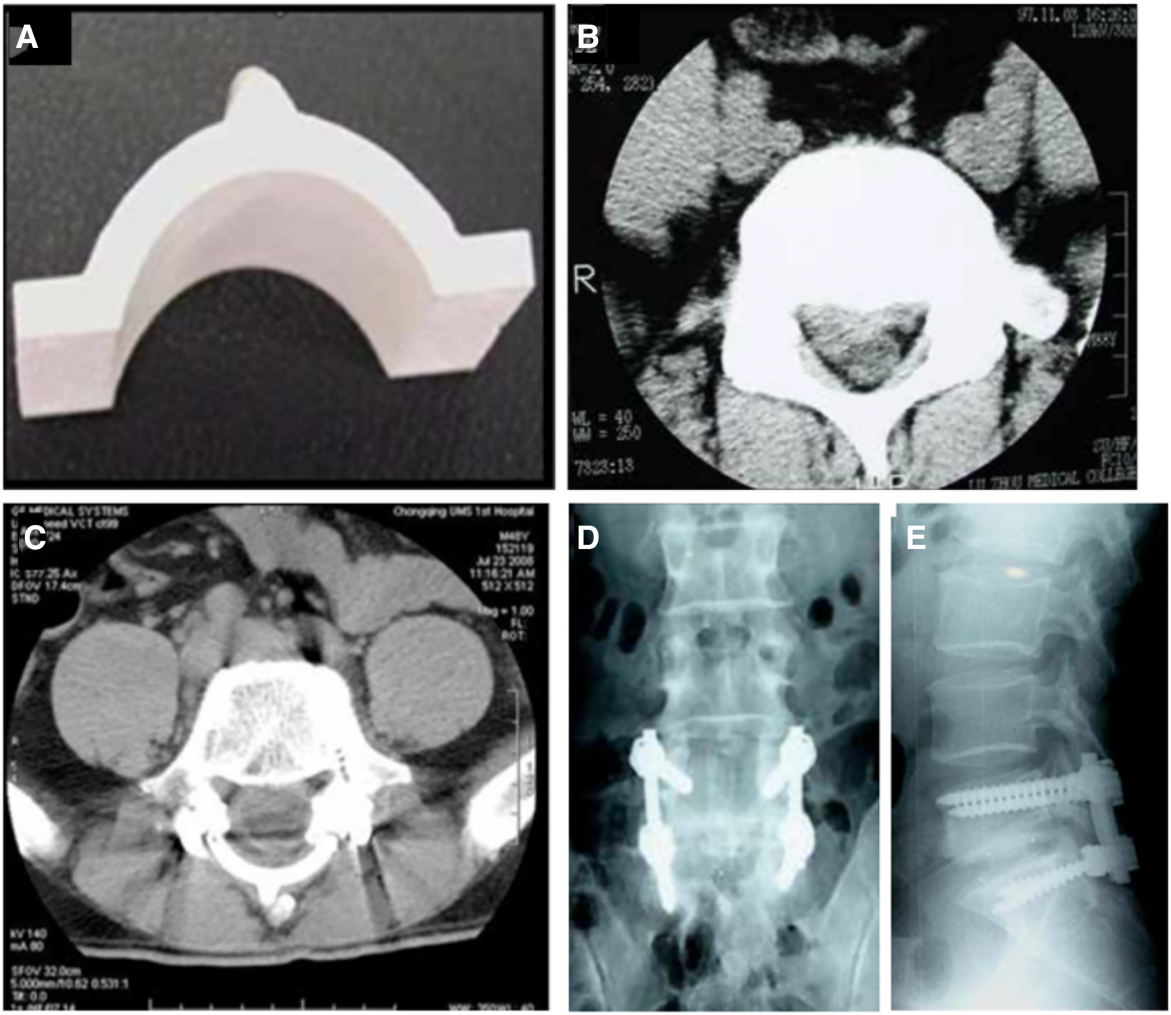
The appearance of the artificial vertebral lamina of n-HA/PA66 composites. Front view (**A**); preoperative CT images showing lumbar disc herniation compressing the nerve root on the right side (**B**). The postoperative CT and radiographs films show good internal fixation with good spinal alignment (**C–E**) (47).

Artificial lamina made of biomaterial is mainly designed to prevent epidural fibrosis. This artificial lamina normally consists of the inorganic element, which provides mechanical support, and the organic element, which prevents scar tissue formation. Zhao et al. (47). designed and fabricated a novel composite artificial lamina made of nano-hydroxyapatite/polyamide66 (n-HA/PA66) in spinal decompression surgery. The n-HA/PA66 composite was developed with a bioactive ceramic (n-HA) and an organic polymer (PA) to mimic natural bone. The n-HA/PA66 material was molded into the hump shape of the lumbar spinal lamina. Eight different scales were designed with 20–35 mm width, 10.6–12.4 mm height, and 4-mm thickness (Figure 1A). After receiving posterior laminectomy and discectomy, patients were implanted with an n-HA/PA66 artificial lamina of appropriate size, which was used to cover the opened spinal canal (Figures 1B, C). The artificial lamina was fixed by titanium rods (Figures 1D, E). At an average follow-up of 5.2 years, the patients showed improved clinical symptoms and significantly improved Japanese Orthopedic Association (JOA) scores. After surgery, the vertebral canal was noticeably enlarged, from 16.7 ± 4.7 mm to 32.9 ± 2.2 mm, and well maintained to 32.1 ± 1.8 mm. The lumbar lordosis was well maintained after surgery. Magnetic resonance imaging (MRI) showed normal spinal canal morphology, with no signs of stenosis, obvious scar formation, or compression of nerve roots or epidural sac. However, the bony union between the host bone and the artificial lamina was not detected, which may lead to the loosening of the pedicle screw and rods. Moreover, the artificial laminae provided limited size options, which hindered its matching with patients’ autologous reserved bony structure.

**Figure 2:**
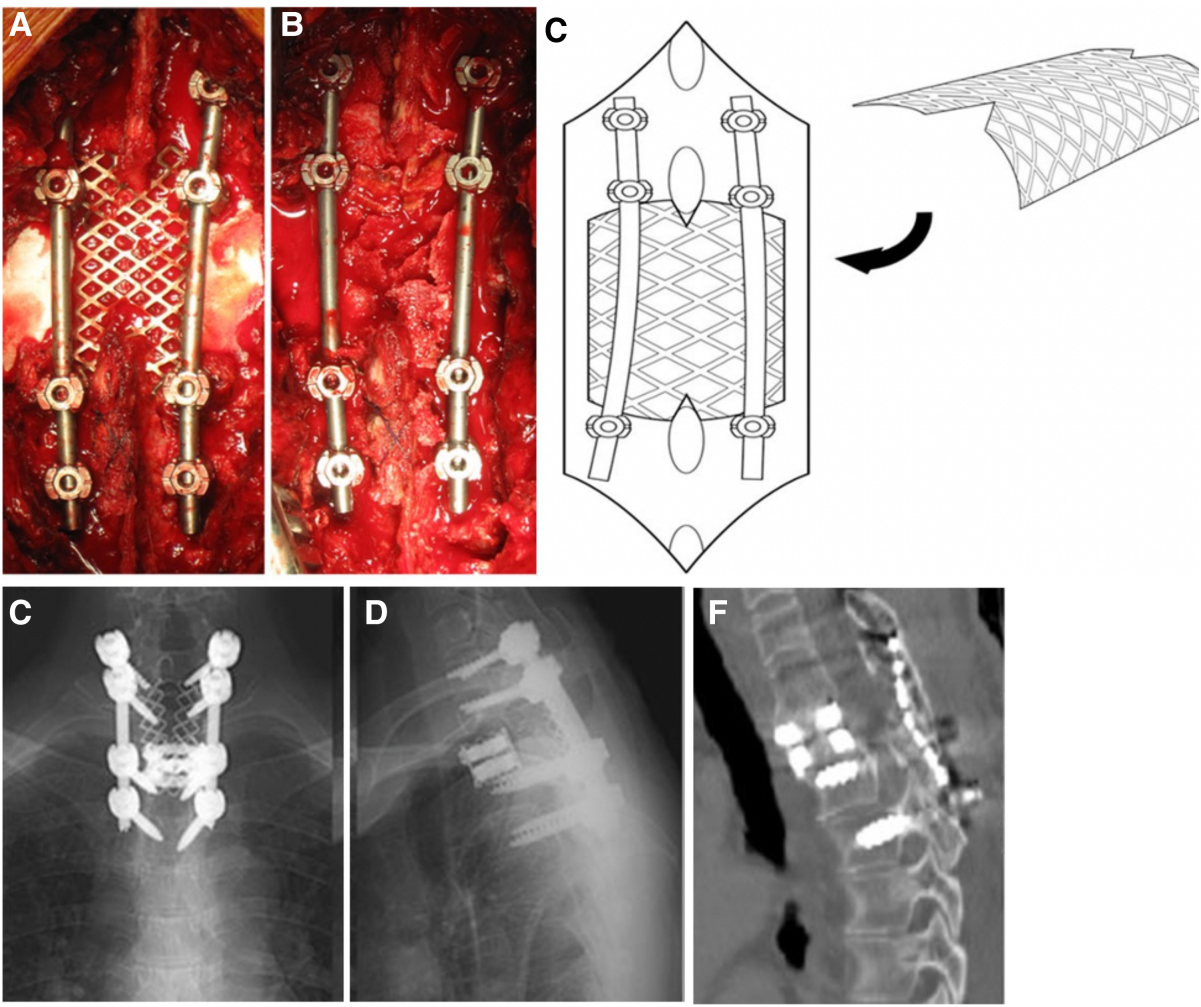
A 55-year-old female with primary T2 vertebra spine tumor (aneurysmal bone cyst) received total *en bloc* spondylectomy. Intraoperative images of the appropriately sized titanium mesh fixed between the upper and lower lamina of total *en bloc* vertebra (**A**). Image of autogenous bone placed on the titanium lamina mesh (**B**). The schematic diagram demonstrated the insertion and fixation of the titanium lamina mesh (**C**). At 28 months of follow-up, radiographs showed no evidence for collapse or displacement of titanium lamina mesh nor instability/malalignment of the spinal column (**D,E**). Postoperative CT image showed complete bony union above the lamina mesh and inside of the titanium MESH cage (**F**) (12).

Compared to artificial lamina made of biomaterial composite, clinical practitioners are more likely to use titanium alloy as implanted prostheses because of its high strength and biocompatibility. It is feasible to obtain postoperative early stability reinforcement, sufficient space and protection for the posterior neural structure, prevention of epidural adhesion, and permanent stability from the new lamina reconstruction after the union of the bone graft. Before the prototype of contemporary artificial laminae used in patients, prostheses used in clinical practice for laminae reconstruction were mainly for laminoplasty. Fornari et al. (16) introduced artificial titanium laminae in 7 patients with progressive and severe myelopathy. Those 7 patients received modified double-door laminoplasty. The double-door laminoplasty procedure was modified by using two artificial titanium laminae which obtained from a simple surgical 0.5-mm Ti-mesh. The sagittal diameter of the cervical spinal canal was reduced (<10 mm) in all cases, and further severe spinal cord compression resulted from osteophytes bars and calcified ligamentum flava at different levels. No abnormal alignment, pathological movements, or instability was present. Postoperative imageological examination demonstrated complete spinal cord decompression and preservation of the patency of the subarachnoid spaces. The proposed laminae have the advantages of achieving immediate stabilization of the spine through a bridge-like mechanism and protection from the possible compression to the dural sac from paravertebral muscles. In another cervical laminoplasty approach, Park et al. (48) introduced a novel titanium plate to maintain canal expansion in multilevel cord compression and cervical myelopathy. The plate afforded an elementary resistance to misplacing the plate or the lamina as the “mouth” of the plate, which accepted the cut edge of the laminae. Moreover, the ventral crotch on the plate's lateral part caught the lamina's cut edge along with it. There were drilling holes and screw insertion facilitated. This novel laminoplasty plate could help minimize the problems with loss of canal expansion and allow the patient to engage in a more aggressive rehabilitation protocol. Furthermore, it could also decrease the incidence of postoperative complications during neck rotation after laminoplasty. To explore the bony reunion effect of artificial titanium lamina in lumbar spine surgery, Chung et al. (12) investigated a new titanium lamina mesh for posterior column reconstruction in patients who underwent total *en bloc* spondylectomy. The prosthesis was an unfolded 1-mm thick titanium mesh. After removing the vertebra body on the posterior side, the surgeon inserted a titanium cage filled with bone graft. Then, the titanium lamina mesh was cut to size according to the resected vertebra level's interlaminar space length and width. The appropriately sized titanium mesh was fixed on vertebral laminae of both upper and lower levels of the spine, along with the autogenous bone (Figure 2). At postoperative 6-month follow-up, there were bony connections between the titanium mesh in both upper and lower adjacent laminae in all cases, except for one with infection. None of the patients had developed collapse or displacement of the implanted lamina or instability/malalignment of the spinal column. The outcome of the titanium artificial lamina implant is encouraging despite the small sample size and short follow-up time. However, this type of artificial lamina could only be used in laminoplasty and could provide no muscle/ligaments attachment; whether it is suitable for lumbar laminectomy remains unclear.

To evaluate the clinical efficacy of different types of artificial lamina, Li et al. (49) compared the clinical efficacy of the simple expansion of the spinal canal decompression, decompression plus hydroxyapatite/polyamide artificial lamina reconstruction, and decompression plus titanium mesh reconstruction in treating spinal canal stenosis. The study found that patients who received artificial lamina reconstruction, hydroxyapatite/polyamide and titanium mesh had better JOA scores than those who underwent a simple decompression procedure in a 12-month follow-up. Despite the small number of studies reported, the authors offered potential and encouraging method to achieve instant and permanent stability of the spine after unionizing the bone graft by artificial lamina implantation. Nevertheless, these artificial laminae have a limited anti-adhesion effect and the biomechanical characteristic remain unclear. Exploring the anti-adhesion mechanism can make the best use of artificial lamina in future clinical practice.

## The progress for the artificial lamina

**Figure 3:**
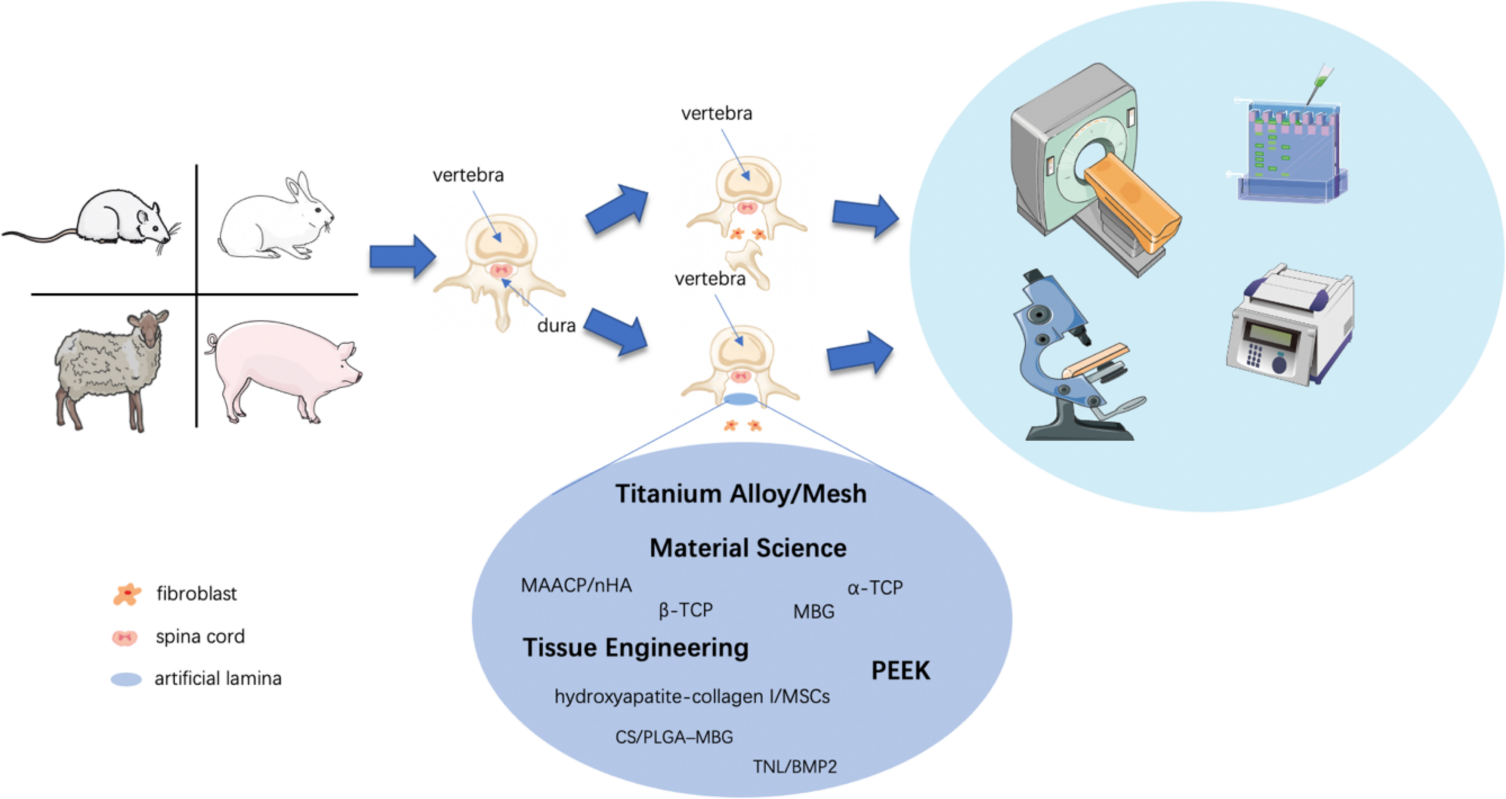
Schematic diagram of techniques investigating artificial lamina in laboratory research. Rats, rabbits, goats, and pigs were sacrificed to form a laminectomy model by lumbar or cervical laminectomy, while the artificial lamina implant model by implantation of various shapes and textures of the artificial lamina. Experimental animals would receive tests, including imageological examinations, histological examinations, western blot, PCR, etc. for biological verification of the artificial laminae.

To prevent postoperative FBSS caused by epidural fibrosis, iatrogenic spinal instability, and muscle weakness, researchers have conducted a lot of research to improve the biomechanical effect and anti-adhesion ability of artificial lamina. Compared to few artificial lamina applications in clinical practice, researchers have undergone many attempts in the laboratory. Because an ideal artificial lamina should offer a mechanical barrier between the dura and the posterior tissue while inducing bony union, which is the ultimate purpose of an artificial lamina prosthesis (Figure 3). Furthermore, the artificial lamina with clarified biomechanical characteristics can stabilize the spine column. Finally, customized prosthesis would have more contact area and better bony union possibility.

### Artificial lamina for epidural fibrosis prevention

Based on the “3-dimensional fibrosis formation theory” proposed by Songer and Ghosh Spencer (39), fibroblast migration from paravertebral muscles and/or movement *via* fluid circulation into the surgical area to form epidural fibrosis, which mechanically tethers and compresses the spinal roots and the neural sac (50). local application of different materials to separate epidural mater from local tissue to prevent fibrosis can be an effective method. Along with the rapid development of materials science and tissue engineering techniques, strategies to prevent post-laminectomy complications are a contemporary research hotspot.

Calcium phosphate has bioinert, good biocompatibility, and mechanical properties, which is currently a significant material for bone tissue reconstruction. Porous calcium phosphate can be fabricated as a scaffold to carry various anti-adhesion components to prevent scar formation (51). Ran et al. (52) used a biodegradable α-TCP/poly (amino acid) composite artificial lamina in goats for the prevention of intraspinal scar adhesion. The results indicated that α-TCP/poly (amino acid) composite artificial lamina might prevent potential scar tissue infiltration into the spinal canal. Wang et al. (53) designed a biphasic calcium phosphate (70% hydroxyapatite/30% tricalcium phosphate) (HA-TCP) ceramic (CVL) implant to evaluate the biological effect after laminectomy in experimental animals. The study showed that fibrous connective tissues and blood vessels had grown into the porous structures of the CVL substitutes rather than into the epidural space, and macrophages were found on the macropore surfaces. After up to 1-year follow-up, CVL could realize the fusion with the natural spine, improve the spinal stability and protect the spinal cord. The study indicates that inorganic material as calcium phosphate and hydroxyapatite were suitable to be a clinical substitute for human vertebral laminae.

Hydroxyapatite (HA), a good biocompatibility and osteoinductivity materials, has been widely used to treat in bone and tooth defects (54–56). Wu et al. (14) discovered that different hydroxyapatite ceramics surface microstructures could prevent the hyperplastic fibrous tissue from penetrating the spinal canal area and inhibited the formation of scar-like tissue in laminectomy sites both *in vitro* and *in vivo* experiments. The artificial laminae were prepared by HA powders with cold isostatic pressing (CIP) and slip casting (SC) technique. Both types of laminae were cut into discs (Φ 9 × 2 mm^3^) and Y-shaped vertebral lamina (10 mm in length, 5 mm in width, and 2 mm in thickness), respectively. *In vitro*, HA ceramics could potentially inhibit the proliferation of the cells and stimulate the CCNI secretion to induce the senescence of human skin fibroblast (HSF) cells. *In vivo*, a dense HA-CIP lamina implant had a better anti fibrosis/adhesion effect, including a thinner layer of fibrous tissue and a smaller gap between the implant surface and paravertebral muscles, than HA-SC in rabbits under laminectomy. These artificial laminae could prevent epidural fibrosis by directly blocking the spinal cord's posterior structure and adjusting the micro-structure to depress the proliferation of fibroblasts.

**Figure 4:**
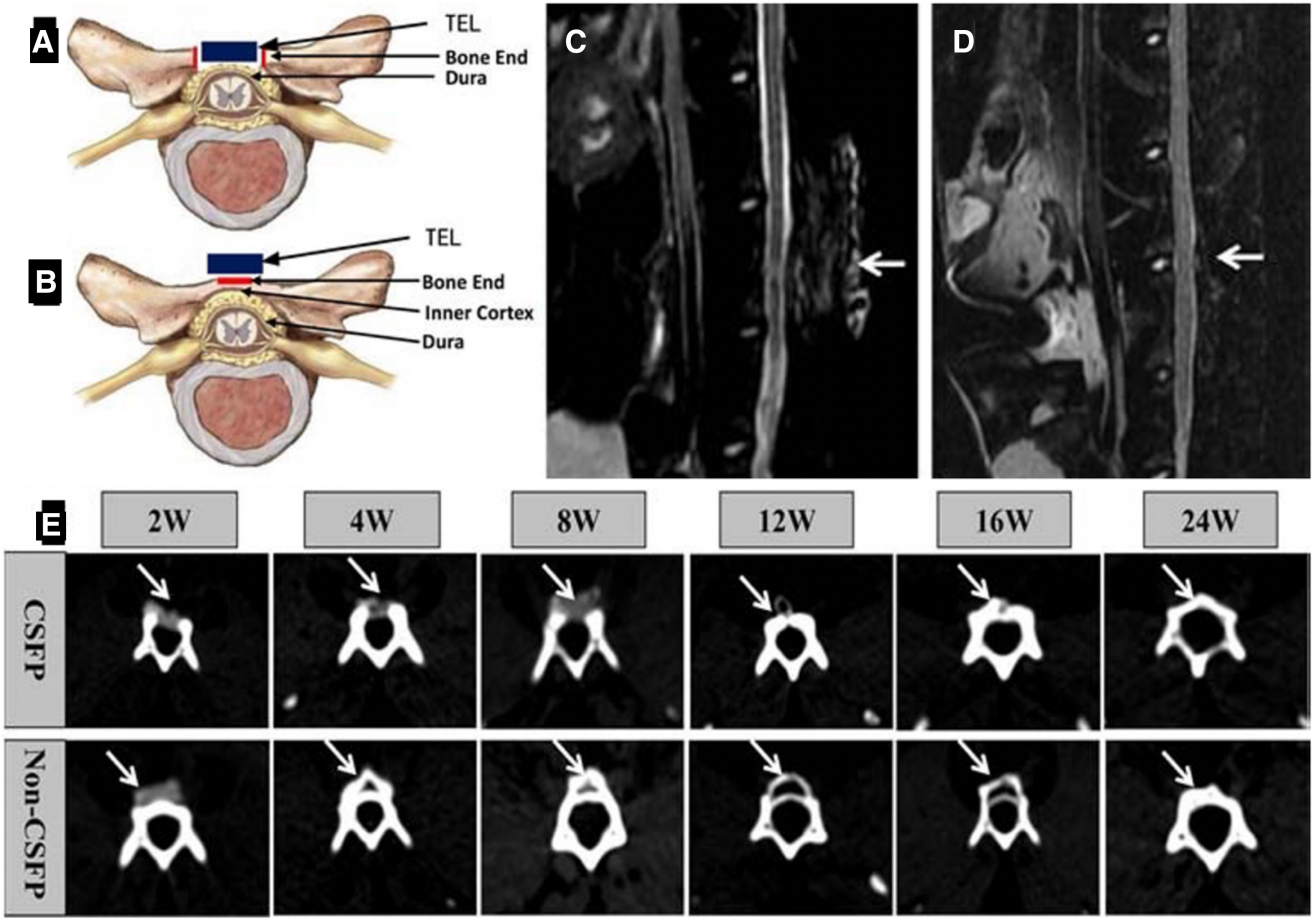
Construction of animal models. (**A**) Diagrammatic sketch of orthotopic laminae animal model. The red square shows the two bone ends, and its length was 2 mm. The blue square shows the tissue-engineered laminae (TEL). (**B**) The diagrammatic sketch of ectopic laminae animal model. The red square shows the bone end, and its length was 4 mm. The blue square shows the tissue-engineered laminae (TEL). MRI scanning of the lumbar spine, 2 weeks after the operation (**C**), 8 weeks after the operation (**D**); white arrow: the targeted vertebrate. (**E**) CT scan of the targeted vertebrate in the 2nd, 4th, 8th, 12th, 16th, and 24th weeks. White arrow: the newborn laminae (57).

Most of the artificial laminae in the laboratory were designed using various inorganic materials, tissue-engineered artificial laminae with added cellular components may be able to act as a better barrier. Li et al. (20, 57, 58) conducted consecutive studies about cerebrospinal fluid pulsation (CSFP) remodeling artificial laminae formation. For the preparation of the artificial lamina, MSCs were obtained from rabbit umbilical cord Wharton's Jelly and their differentiation into osteogenic MSCs was induced using a hydroxyapatite-collagen I scaffold. The tissue-engineered lamina (TEL) scaffold was cut into the size of 10 mm × 8 mm × 20 mm (57). The experimental CSFP rabbits received open posterior lumbar 5 laminectomies under anesthesia. Then, the TEL was placed on a 10 mm × 8 mm × 2 mm bone defect and fixed and sutured. The spinous processes of non-CSFP rabbits were removed from the top of the process to the cancellous bone end while preserving the dura surface cortex of the laminae (Figure 4B). The TEL was then fixed onto the native laminae (20). MRI examination showed no signs of epidural scar adhesion, spinal cord compression, or intervertebral disc herniation in the targeted vertebrae or adjacent segments in both CSFP and Non-CSFP groups (Figure 4). Similar to the studies mentioned above, these studies did not perform the osteogenesis effect of the artificial lamina. Therefore, it is not certain that the structure will remain stable between the regeneration of the new bone and the degradation of the implanted composite. The artificial lamina could have a promising effect in dealing with the epidural fibrosis problem. On the other hand, the osteoinductive effect of the artificial lamina is determinant since bony reconstruction is the most stable structure for the long term.

### Artificial lamina for osteogenesis induction

Artificial lamina could be implanted not only as a barrier to prevent epidural fibrosis but also as a “bridge” for osteogenesis induction and bony reconstruction of the posterior column. Many materials with osteoinductivity are used to fabricate artificial lamina. Nowadays, calcium phosphates are mainly used to induce the repair or regeneration of damaged bone tissue (51). β-TCP had been applied as a supplement lamina autograft to perform posterolateral lumbar-instrumented arthrodesis before being fabricated as a lamina shape, (11). Calcium phosphates can be implanted alone and serve as a scaffold for other bio-materials. Dong et al. (59) used bone marrow mesenchymal stem cells (BMSCs) to reconstruct laminae in rabbits. The BMSCs were seeded into porous β-TCP bio-ceramics and cultivated with osteogenic supplements for less than 3 weeks. Rabbits received L5/L6 laminectomy with a bone defect measuring approximately 20 mm × 8 mm. After 8 weeks’ observation, rabbits that received β-TCP bio-ceramics implanted with BMSCs showed signs of regeneration of the lamina of the vertebral arch. Imaging examinations (CT and MRI) at 16 weeks showed the successful formation of the artificial lamina of the vertebral arch. In another research conducted by Dong et al. (60), the authors induced osteoblastic differentiation of BMSCs, which were then transplanted into collagen sponges to construct the tissue-engineering bone. After receiving laminectomy and implanted with collagen sponge and tissue-engineered bone, the rabbits were found to successfully artificial laminae of the vertebral arch formation 4 weeks after the operation. This tissue engineering designed artificial lamina seems a promising method for future mass production.

**Figure 5:**
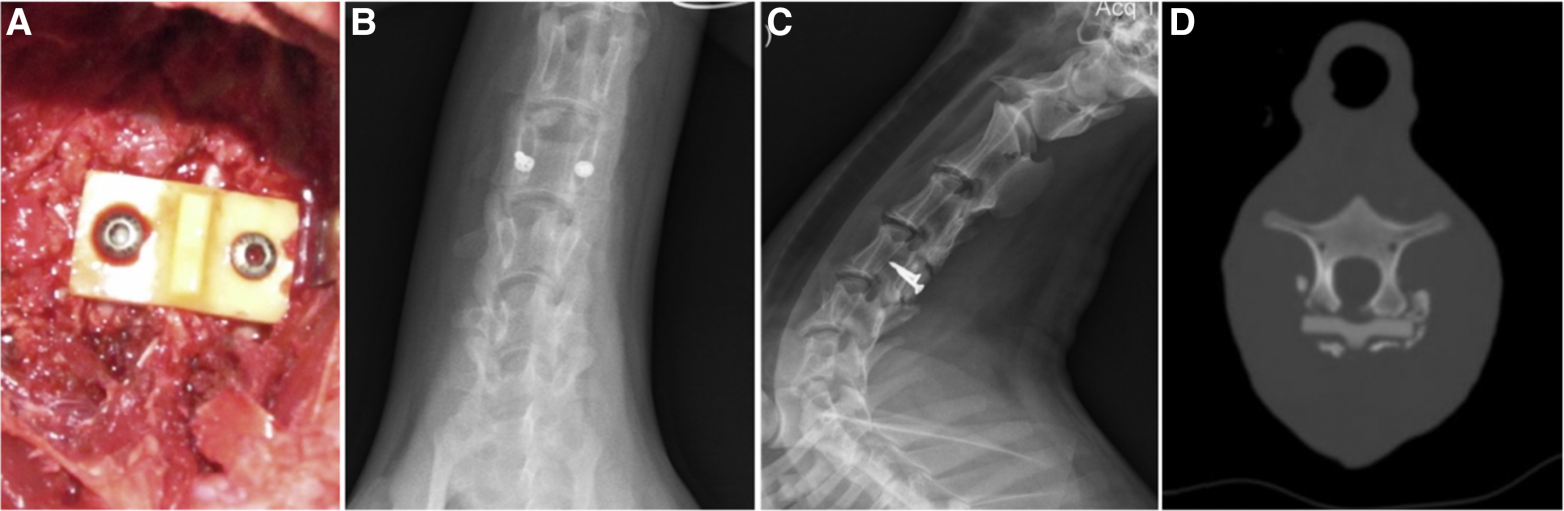
A biodegradable MAACP/nHA composite artificial lamina was implanted to cover the dura before fixing it on the pedicle cervical 4 *via* 2 screws. Insertion of the lamina (**A**). Radiograph and CT images of goat cervical from the test group received biodegradable MAACP/nHA composite artificial laminae after 24 weeks. (**B–D**) The artificial lamina was used to cover a defect, and no artificial lamina displacement was found in the test group. (**B**) longitudinal proliferation along the edge of the artificial lamina is observed without the C4 spinous process. (**C**) CT image shows new bone formation above the artificial lamina. (**D**) (61).

The porous HA is another important material as a scaffold for bone tissue regeneration. It may act as a solid matrix for adsorption, storage and controlled release of circulating or locally produced bone morphogenetic proteins, which locally initiate bone formation (55). In a study related to the utilization of hydroxyapatite, Lv et al. (61) introduced a novel biodegradable artificial lamina, which was made of multi-amino acid copolymer/nanohydroxyapatite (MAACP/nHA) copolymer composite. This artificial lamina was designed to prevent epidural adhesion surrounding the bony defect and promote bone tissue repair. This artificial lamina composite was a hard nHA material (strength: 257.53 MPa, yield strength: 42.77 MPa, and modulus: 350 MPa), which could provide enough strength to prevent dural compression by the posterior tissue. The artificial lamina was a smooth rectangular plate (28 mm × 16 mm × 4 mm) with a natural lamina-like spinous process (Figure 5A). In the experimental study, cervical 4 laminae of goats were removed to create 27 mm × 9 mm bone defects without damaging the small facet joints. Then, the biodegradable composite artificial laminae were implanted to cover the dura before fixation on the pedicle cervical 4 *via* 2 screws. Postoperative images showed no displacement of the composite or dural adhesion compression (Figures 5B, C). An imaging examination performed 24 weeks after the operation showed new cervical natural bone in the defect forming the reconstructed bony spinal canal (Figure 5D). This study has put the mechanic module of artificial lamina into practice. However, no analysis of the biomechanical test between these cervical composite laminae and autologous bone was performed.

CS/PLGA–MBG is widely used as a porous scaffold in tissue engineering applications. For one, the porous structure of the scaffold can load organic material, such as MSCs, to induce osteogenesis; on the other hand, new bone tissue can grow into porous structure to promote bone fusion. In another study of artificial lamina developed by tissue engineering, Han et al. (14) designed a Chitosan (CS)/poly lactide-co-glycolide (PLGA) bilayer membrane loaded with tranilast (TNL) and BMP-2. TNL is an oral anti-allergic drug that has been shown to be highly effective in various fibrotic disorders by inhibiting collagen deposition and fibroblast proliferation. MBG, which has a mass of porosity and interconnected pore network, has been shown to enhance human MSCs infiltration, attachment, and proliferation and promote osteogenic differentiation. This bilayer composite was implanted as an artificial lamina into the rabbit with an L5 lamina bony defect. Compared to the control group, there was no fibrous tissue formation or invasion in the TNL/BMP-2 experimental group. Moreover, the new bone formation in the experimental group at the laminectomy site was loaded with TNL/BMP-2. This multifunctional bilayer composite seems to be an ideal artificial lamina. Nevertheless, the complex methodology for preparing the bilayer membrane hinders its mass production for clinical application.

Different artificial laminae's biological effects have been reviewed, including epidural fibrosis prevention and osteoinductivity. However, the human spine column is a load-bearing system, and the biomechanical effect is also very important.

### Artificial lamina with biomechanical effect

Despite the wide application of biomaterials and drugs for the prevention of epidural fibrosis (62–65), the beneficial effects are not sustained, and the effect decreases over time. Some of the artificial laminae mentioned above have a biomechanical effect, but none of them have clarified the stress distribution of the implant and the spine. The artificial laminae applied in clinical practice merely provides mechanical support, for which no biomechanical analysis was performed. Therefore, the postoperative stress on the spine and the prostheses is unknown, which may lead to further complications. To explore the artificial lamina's biomechanical effect, interdisciplinary medical engineering, such as finite element analysis (FEA) and topological optimization (TO), have been introduced. By using finite element analysis, the biomechanical effect of the whole postoperative spine complex can be analyzed, and the artificial lamina can be topologically re-designed to a perfect state.

Compared to many FEA studies on vertebral bodies and intervertebral discs, fewer vertebra lamina FEA studies have been conducted. Most of them focused on analyzing various surgical approaches for posterior decompression. Spina et al. (66) investigated the effect of graded pars interarticularis (PI) resection in a three-dimensional manner on PI stress to provide surgical guidelines to avoid iatrogenic instability following lumbar laminectomy using a biomechanical finite element method. Based on a series of finite element analyses, the authors concluded that PI resection exceeding 50% bone resection greatly reduces the spine's stability for all laminectomies and may increase the incidence of iatrogenic spondylolisthesis. The study demonstrates that the biomechanical properties of vertebral lamina is very important, and it is necessary to reconstruct the mechanical function of lamina.

**Figure 6:**
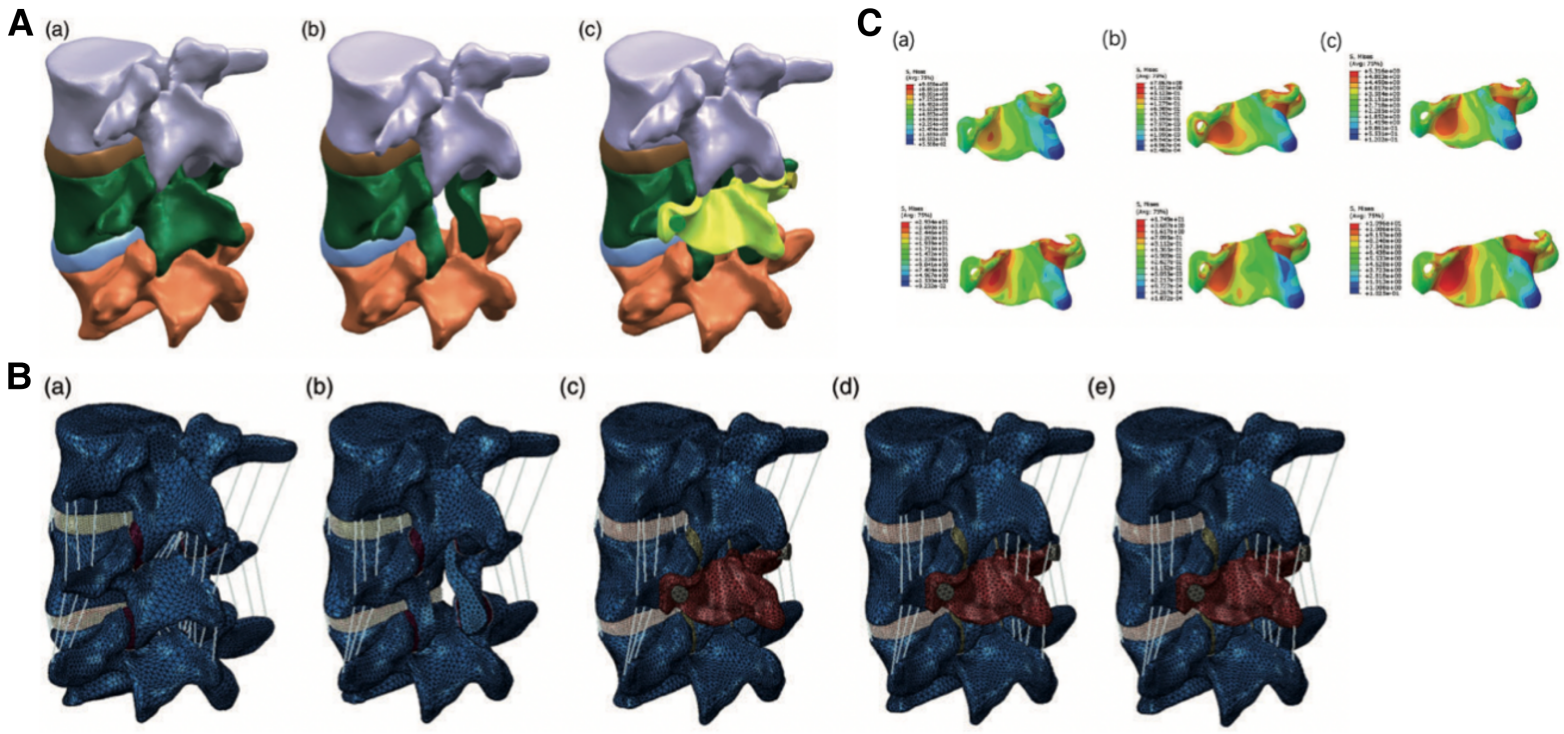
(**A**) the artificial lamina model of L4. (**a**) Intact lumbar spine; (**b**) Laminectomy model; (**c**) Laminectomy model with artificial lamina (yellow part). (**B**) The finite element model of different surgical stages. (**a**) Intact lumbar model; (**b**) Laminectomy model (LN); (**c**) Artificial lamina alone model (ALA); (**d**) Artificial lamina with ligament reconstruction model (ALR); (**e**) Artificial lamina with osseointegration model (ALO). (**C**) Stress distributions in the PEEK artificial lamina in all motions. Stress concentration in the area corresponding to the in-situ lumbar isthmus. (**a**) flexion and extension stress distribution in the ALA model; (**b**) flexion and extension stress distribution in the ALR model; (**c**) flexion and extension stress distribution in the ALO model (68).

In a recent artificial lamina designing study developed by FEA, Liu et al. (67) used poly-ether-ether-ketone (PEEK) to establish the system, which could stabilize the lumbar spine and prevent postoperative spine malfunctions such as ASD and iatrogenic lumbar deformities. The researchers derived a finite element model of L3–5 from computed tomography images (Figure 6A). Apart from the intact human spine model, 4 surgical models, including laminectomy, artificial lamina alone, ligament reconstruction, and osteointegration, were constructed (Figure 6B). The 4 surgical models were used to simulate 4 different stages of L4 artificial lamina implantation. FEA of the artificial lamina showed that the artificial lamina could stabilize the lumbar isthmus and share the stress after laminectomy (Figure 6C). These findings suggested that the PEEK artificial lamina may have the potential to stabilize the post-laminectomy lumbar spine and prevent related complications.

FEA can clarify the mechanical contribution of the artificial lamina and demonstrate the mechanical distribution between the prosthesis and the autologous spine, which guide the artificial lamina design for posterior column reconstruction of the spine.

### Artificial laminae for personal customization

Lumbar spine after laminectomy should be biomechanically stable, especially multi-level laminectomy requires artificial lamina reconstruction. Additive manufacture (AM), also known as 3D printing, can provide a viable alternative for producing artificial lamina. 3D printing is based on computer-generated three-dimensional images, which can accurately manufacture a variety of physical structures. This technology has been applied to industrial design, architecture, engineering, automotive, and aerospace fields. In medicine, 3D printing is applied for preoperative planning and design of surgical instruments and individualization of prostheses, etc. (69–71). The application of 3D printing technology in the field of spine surgery had obtained enormous and substantial progress. Among which, vertebral skeleton model (including lesion model) printing has been widely used in clinical application due to its relatively simple technology and low cost (72). The part with full expectation is undoubtedly the clinical application of 3D printing microporous metal implant and personalized implant as well as the clinical application of 3D printing biological materials in the future (73). Many bio-and/or non-biomaterials have been used to make artificial laminae, as described above. Among those materials, titanium (Ti) alloy has favorable mechanical property features and is widely used in clinical fields (74–77).

The combination of computational design optimization with 3DP technologies allows for the realization of architecture optimized custom-designed implants and opens the way to promising future surgical solutions (78). A recent study yielded encouraging results of 3D printing for artificial lamina design. Li et al. (79) designed an individualized titanium alloy spine lamina using 3D printing technology and evaluated its effectiveness by implantation in human cadaveric spines. The authors used computed tomography (CT) to reconstruct the lumbar vertebrae and simulating lumbar laminectomy. Then an artificial lamina was designed to fill the post-laminectomy bone defect. The lamina made of titanium alloy was fabricated by 3D printing in the shape of the native lamina, which included edge passivation, thinning of the lamina thickness of the spinous process root, and increasing the curvature of the ventral lamina to increase the spinal canal volume. This artificial lamina also involved attachment holes to fix paravertebral muscles at the spinous process. One advantage of this lamina was the statistically significant enlargement of the bony canal after laminectomy compared to before surgery (356.17 ± 43.11 mm^2^ vs. 311.23 ± 38.17 mm^2^). In a further study conducted by the same team (25), a novel type of 3D-printed bionic titanium alloy artificial lamina was fabricated and implanted into a pig laminectomy model. *In vitro* and *in vivo* tests have shown good biomechanical effect and well fixation of bionic titanium alloy artificial lamina and screws 10 weeks after laminectomy. This 3D printed artificial lamina can prevent epidural adhesion while restoring the structural stability of the posterior complex, suggesting the potential of lamina substitutes for adhesion prevention after laminectomy.

Artificial lamina under AM is accurately manufactured as a variety of physical structures accordance with the native lamina functional parts. This prosthesis not only has biological characteristics, but also can improve the biomechanical structure according to the results of mechanical analysis. Some barriers of 3D printing artificial lamina in widespread adoption include financial burden (both on the hospital and the patients) and time consuming. And only very few specialists can apply this method in clinical practice (80). Despite the limitations of 3D printing in clinical adoption, patients would benefit more when 3D printing technologically develops and the cost reduces. Therefore, as clinical practitioners, we should do better designing and demonstrating, preparing for future mass adoption.

## Complications of artificial lamina and limitations

Complications of artificial lamina include infection, bone nonunion and the unparralled of osteogenesis and biomaterial degradation.

Postoperative infection is a major complication of artificial lamina implantation. Jae-Yoon Chung et al. (12) investigated a new titanium lamina mesh for posterior column reconstruction in patients who underwent total *en bloc* spondylectomy. The prosthesis was an unfolded 1 millimeter thick, titanium mesh. One of the eight patients suffered postoperative infection at the surgical site. To solve this complication, Liu et al. investigated a surface-modified 3D printed porous Ti6Al4V possesses balanced antibacterial and osteogenic functions and found well outcome (73). Bone nonunion is another major complication in prosthesis implant surgeries. To prevent nonunion and promote bony reunion, artificial laminae for osteogenesis induction have been investigated, which were described above. Finally, despite the widely application of biomaterials and drugs for prevention of epidural fibrosis and promote osteogenesis, the duration of effect is unstable and the effect is decreasing over time. The proportion of osteogenesis contained and the speed of its release need more experimental and further study. The unparalleled of osteogenesis and biomaterial degradation is another risk factor of bone nonunion (81).

We have reviewed different types of artificial lamina with various functions, yet clinical trials or randomized control tests (RCTs) are not available to prove the clinical evidence of artificial lamina, further studies are necessary for these issues.

## Discussion

Lumbar laminectomy is a commonly used procedure for spinal decompression in routine clinical practice. In patients with spinal disorders, laminectomy is often performed as a spinal decompressive intervention. However, the loss of posterior column bony support, the extensive proliferation of fibroblasts and scar formation after laminectomy, and other complications (such as postoperative epidural fibrosis and iatrogenic instability) may cause new symptoms requiring revision surgery. Given the inevitable surgical intervention of posterior open decompression procedure in some critical spine disorders, artificial lamina prosthesis implantation is one of the most important methods to avoid post-laminectomy complications. An ideal artificial lamina should not only serve as a physical barrier between the dura mater and the posterior tissue but also provide mechanical support to the posterior spinal column. The combined use of FEA and 3D printing can improve the practicability and clinical application of artificial lamina. However, the accuracy of the algorithm and the 3D printed material needs to be demonstrated. Using FEA and topology optimization for titanium 3D printing, the stress distribution of lumbar vertebrae can be analyzed, and the real stress can be simulated. 3D printing technology allows the design and manufacture of microporous structures with low elastic modulus. Finite element analysis of the individualized anatomical shape lumbar lamina has been carried out to verify the macro biomechanical advantages of the individualized anatomical bionic shape lumbar artificial lamina. Customized titanium artificial lamina has the potential to prevent epidural fibrosis and can reconstruct biomechanical structure, providing adequate dynamic support.
